# Lipids characterization of ultrasound and microwave processed germinated sorghum

**DOI:** 10.1186/s12944-017-0516-4

**Published:** 2017-06-27

**Authors:** Sadia Hassan, Muhammad Imran, Nazir Ahmad, Muhammad Kamran Khan

**Affiliations:** 1 Department of Food Science, Nutrition & Home Economics, Government College University, Faisalabad, 38000 Pakistan; 2 Institute of Home and Food Sciences, Faculty of Science and Technology, Government College University, Faisalabad, 38000 Pakistan

**Keywords:** Sorghum, Germination, Microwave, Ultrasonic, Oil content, Fatty acid composition

## Abstract

**Background:**

Cereal crops and oilseeds provide diverse pool of fatty acids with characteristic properties. Sorghum (*Sorghum bicolor* (L.) *Moench*) provides the staple food with serving as main source of energy and protein. Germination of sorghum generally increases the nutritive value of seeds and the effects of germination on lipids composition of seeds vary greatly with processing conditions. Therefore, the current study was conducted to compare the effect of emerging processing techniques such as ultrasound (US) and microwave (MW) on fatty acids composition and oil yield of sorghum seeds before and after germination.

**Methods:**

Initially sorghum grains were soaked with 5% NaOCl (sodium hypochlorite) for surface sterilization. Afterwards, grains were soaked in excess water for 22 h at room temperature and were divided into four portions. The first portion (100 g grains) was subjected to germination without applying any microwave and ultrasonic treatment (T_0_). Second portion was further divided into four groups (T_1_, T_2_, T_3_, T_4_) (100 g of each group) and grains were subjected to ultrasonic treatments using two different ultrasonic intensities (US_1_: 40%; US_2_: 60%) within range of 0–100% and with two different time durations (t_US1_: 5 min; t_US2_: 10 min) at constant temperature. Third portion was also divided into four groups (T_1_, T_2_, T_3_, T_4_) (100 g of each group) and exposed to microwave treatments at two different power levels (MW_1_: 450 watt; MW_2_: 700 watt) within the range of 100-900 W for two different time durations (t_MW1_: 15 s; t_MW2_: 30s). Similarly, fourth portion was divided into four groups (T_1_, T_2_, T_3_, T_4_) (100 g of each group). Each group was exposed to both MW (MW_1_, MW_2_) (100–900 watt power) & US (US_1_, US_2_) (0–100% intensity) treatments at two different time levels (t_US_, t_MW_). Then, germination was carried out and pre-treated raw and pre-treated germinated sorghum grains were analyzed for total oil yield, fatty acid composition and unsaturated fatty acids (Un-SFA)/saturated fatty acids (SFA) ratio by gas chromatography.

**Results:**

The results revealed that oil yield in sorghum before and after germination ranged from 6.55 to 7.84% and 6.28 to 7.57%, respectively. All the microwave and ultrasound processed samples showed significant difference in oil yield than the raw sorghum grains. The highest tested yield was 7.84 ± 0.31% when combination of microwave power (700 W) and ultrasound intensity (60%) was applied for 30s and 10 min, respectively. The results further demonstrate that the raw sorghum contained palmitic (13.73 ± 0.10%), palmitoleic (0.43 ± 0.02%), stearic (1.07 ± 0.04%), oleic (37.15 ± 0.10%), linoleic (43.33 ± 0.21%), linolenic (1.55 ± 0.04%), arachidic acid (0.13 ± 0.01%) and eicosenoic acid (0.37 ± 0.02%), respectively. The highest fatty acid percentage for palmitic, stearic and arachidic acid was 13.75 ± 0.07%, 1.11 ± 0.09% and 0.15 ± 0.03% at 60% US intensity for 10 min (T_4_), respectively. Maximum amount observed was 1.60 ± 0.09% of linolenic acid while amount of eicosenoic acid decreased from 0.37 ± 0.02% to 0.31 ± 0.01% after processing. In case of applying combination of microwave and sonication treatments, the change in eicosenoic acid increased from 0.35 ± 0.02% to 0.40 ± 0.04% while there was no significant change in other fatty acids. The ungerminated sorghum oil possessed 14.93–15.05% and 82.83–83.12% of SFA and Un-SFA, respectively. After germination, percentage of saturated fatty acids increased (16.4–16.55%) while decreased for unsaturated fatty acids (80.13–80.56%) were noted.

**Conclusions:**

The results of the present study conclude that the yield of oil from sorghum grains increased by emerging processing. Fatty acid analysis of sorghum oil suggested that pre-treatment strategies will not affect the quality of the oil with respect to essential fatty acids content. Overall, the composition of saturated fatty acid in germinated grain is improved than ungerminated grains after processing.

## Background

Lipids as constituent of diet and may perform essential role regarding human health and disease prevention [1]. They perform a variety of functions in a biological system as source of energy, essential fatty acids, sterols, structural components of membranes, transport medium of metabolic fuel, provide protective covering and carriers of lipophilic vitamins. Dietary lipids perform regulatory actions in nutrient metabolism and cell functions through controlling gene expression [2]. Such regulatory lipids have been categorized as “functional lipids” including omega-3 and omega-6 fatty acids, conjugated linoleic acids, medium chain triglycerides and phytosterols. These lipids have many beneficial effects on human health such as in obesity, bone health, managing depression, blood pressure and cardiovascular health [1].

Cereal grains such as wheat, rice, maize, sorghum, millet, barley and rye in their natural form (as whole grain) are rich source of vitamins, minerals, carbohydrates, fats, oils and protein [3]. Whole grain cereals containing nutrients and bioactive substances have health-promoting effects and the evidence for this association is largely supported by observational studies [4]. The consumption of three or more servings of whole grains is associated with a positive impact on body mass index (BMI), abdominal obesity, cardiovascular disease risk reduction and glucose homeostasis [5]. For centuries, germination process has been used for the purpose to soften the grain structure, improvement in nutritional value, anti-nutritional compounds reduction and enhancement in functionality of seed components [6, 7]. Seed germination causes different biochemical activities thus resulting in chemical composition changes [8]. In the last decades, experts dealing with the healthy nutrition turned their attention towards the determination of the biological value of the nutritional sprouts [9]. The improved food value of sprouting grains has been used for human consumption in Asian countries [10]. Besides the nutrients, sprouts are also considered as the source of phytochemicals, vitamins, minerals, amino acids and enzymes for contribution in improved human health [11].

To improve bioevaluation and bioavailability performance, seed pretreatments including physical and chemical treatments are widely used. Physical treatments such as irradiation and electrical are known to improve seed performance and germination [12]. During germination, lipids, carbohydrates and proteins within the seed are broken down into essential compounds while some nutrients also transform to bioactive components [13]. However, among the different emerging novel techniques of pre-treatment gaining interest such as ultrasound (US) and microwave (MW) assisted processes are playing the leading role. The use of US and MW assisted process reduces energy consumption and also favors safe, robust and controlled processes [14]. Therefore, the main mandate of the research was to determine the effect of US and MW processing conditions on fatty acids composition and oil yield of sorghum seeds before and after germination.

## Methods

### Raw materials

Sorghum (*Sorghum bicolor* (L.) Moench) grains selected for this study were purchased from a local grain market of Faisalabad (Pakistan). Sorghum grains were cleaned to remove stones, dust glumes, stalks, light materials, broken, undersized and immature grains. Cleaning was done by hand sorting and winnowing. Sorted and cleaned grains were kept in high density polyethylene to avoid moisture uptake and contamination before use.

### Seed treatments

Grains were soaked with 5% NaOCl (sodium hypochlorite) for surface sterilization upto 5 min to avoid fungal invasion, followed by washing with distilled water until they reached neutral pH [15]. Afterwards, grains were soaked in excess water for 22 h at room temperature. The steeping water was drained off and the soaked sorghum grains were washed twice using distilled water. The soaked sorghum grains were divided into four portions.

#### Control

The first portion (100 g grains) was subjected to germination without applying any microwave and ultrasonic treatment (served as control: T_0_).

#### Ultrasonic (US) treatment

Second portion was further divided into four groups (T_1_, T_2_, T_3_, T_4_) (100 g of each group) and grains were subjected to ultrasonic treatments using two different ultrasonic intensities (US_1_: 40%; US_2_: 60%) within range of 0–100% and with two different time durations (t_US1_: 5 min; t_US2_: 10 min) at constant temperature [16]. All ultrasonic treatments were carried out through ultrasonic processor of SONICS & MATERIALS. INC (model: VCX750) having power of 750 W, frequency 20 kHz and volts 230 VAC ~ 50/60 Hz NOM.

#### Microwave (MW) treatment

Third portion was also divided into four groups (T_1_, T_2_, T_3_, T_4_) (100 g of each group) and exposed to microwave treatments at two different power levels (MW_1_: 450 watt; MW_2_: 700 watt) within the range of 100-900 W for two different time durations (t_MW1_: 15 s; t_MW2_: 30s) [17]. All microwave treatments were carried out with HOMAGE microwave oven (model: HDSO234S) having capacity of 23 L with rated voltage of 230 V~, rated frequency 50 Hz, rated input 1250 W, rated output 800 W and microwave frequency of 2450 MHz.

#### US & MW treatment

Similarly fourth portion was also divided into four groups (T_1_, T_2_, T_3_, T_4_) (100 g of each group). Each group was exposed to both MW (MW_1_, MW_2_) (100-900 W power) & US (US_1_, US_2_) (0–100% intensity) treatments at two different time levels (t_US_, t_MW_).

The detailed treatment plan has been presented in Table 1.

**Table 1:** Ultrasound and microwave treatment layout for sorghum grains

Treatments	Processing type					
	Ultrasonic treated (sprouts) B_1_		Microwave treated (sprouts) B_2_		Ultrasonic & Microwave treated (sprouts) B_3_	
	Time t_US_ (min)	US Intensity (%)	Time t_MW_ (sec)	MW Power (watt)	Time t_US_ + t_MW_	US Intensity (%) + MW Power (watt)
Raw seed	----	----	----	----	----	---------
T_0_	----	----	----	----	----	---------
T_1_	t_US1_ 5 min	US_1_ 40%	t_MW1_ 15 s	MW_1_ 450 W	t_US1_ + t_MWI_ 5 min + 15 s	US_1_ + MW_1_ 40% + 450 W
T_2_	t_US1_ 5 min	US_2_ 60%	t_MW1_ 15 s	MW_2_ 700 W	t_US1_ + t_MWI_ 5 min + 15 s	US_2_ + MW_2_ 60% + 700 W
T_3_	t_US2_ 10 min	US_1_ 40%	t_MW2_ 30s	MW_1_ 450 W	t_US2_ + t_MW2_ 10 min + 30s	US_1_ + MW_1_ 40% + 450 W
T_4_	t_US2_ 10 min	US_2_ 60%	t_MW2_ 30s	MW_2_ 700 W	t_US2_ + t_MW2_ 10 min + 30s	US_2_ + MW_2_ 60% + 700 W

*T*_*0*_ *: Control germinated sample; T*_*1*_ *, T*_*2*_ *, T*_*3*_ *, T*_*4*_ *: Different treatments*

*MW*_*1*_ *, MW*_*2*_ *: Microwave power levels; US*_*1*_ *, US*_*2*_ *: Ultrasonic intensity levels*

*t*_*US1*_ *, t*_*US2*_ *, t*_*MW1*_ *, t*_*MW2*_ *: Time duration levels*

### Germination and fat extraction

To conduct germination all the untreated (control sample) and treated grains (100 seeds of each group) were subjected to germination. Grains were placed on moist paper towel sheet and covered with another sheet of paper towel. The germination trays were placed in an incubator at 25 ± 2 °C, germinated for 48 h and watered 2–3 times a day to enhance the germination process [18]. The seed was considered to have germinated when both the plumule and radicle had emerged >0.5 cm [19].

After germination, raw seed, control germinated sample and all treated samples were washed using running distilled water and dried in a drying oven at 55 °C for 24 h then pulverized into a fine powder by using a stainless steel blender. The oil was removed from the sample using a Soxhlet apparatus (hexane 8 h). Two different treatments were used to extract the fat: in the first, the fat was extracted directly from the powder, while in the second the powder was hydrolyzed using an aqueous solution of HCL 6 N in reflux during 8 h before the extraction with the hexane [20]. The oil samples were stored at 4 °C in an amber bottle. Percentage of seed oil yield was calculated as follows.

Yield of seed extract (%) = (Oil extract from seeds (g) / Initial weight of seeds (g)) × 100.

### Total fatty acid analysis

The fatty acids profile of extracted oil samples was determined by the method Ce 1f–96 given in AOCS [21]. The oil sample (50 μL) was methyated in the presence of 4 mL KOH (1 M) at room temperature for 1 h in order to convert fatty acids into their respective methyl esters. The resultant fatty acid methyl esters (FAMEs) were extracted with GC grade n–hexane and analyzed by Gas Chromatograph apparatus equipped with an auto sampler, flame–ionization detector (FID) and supelco wax column. The samples (1 μL) were injected with Helium (1 mL/min) as a carrier gas onto the column, which was programmed for operating conditions such as column oven temperature 160 °C at 0 min with subsequent increase of 3 °C/min until 180 °C. The column oven temperature was increased from 180 °C to 220 °C at 1 °C/min and was held for 7.5 min at 220 °C. Split ratio was 50% with injector 240 °C and detector 250 °C temperatures. The peak areas and total fatty acids composition were calculated for each sample by retention time using Varian Chem Station software. The standards of fatty acids methyl esters purchased from Sigma-Aldrich were also run under the same conditions for comparison with experimental samples.

### Statistical analysis

The data of oil yield and fatty acids composition obtained for each treatment was subjected to statistical analysis to determine the level of significance by using the software package (Statistic 8.1) according to the method described [22]. The average of the three runs was reported as the measured value with standard deviation. The Duncan’s multiple range (DMR) test was used to estimate the level of significance that existed between the mean values at a probability level of 5%.

## Results

The average oil content in raw sorghum was found 6.55 ± 0.25%. The oil extraction yield before and after germination by applying different processing pre-treatments have been presented in Table 2. It was found that oil extraction yield was increased slightly when microwave pretreatment power level and time was increased. The optimum condition for microwave pretreatment was 450 W (T_3_) and 700 W (T_4_) which resulted in the highest oil yield 7.54 ± 0.31% and 7.79 ± 0.32%, respectively (*p* ≤ 0.05). Ultrasonic intensity 40% (T_3_) and 60% (T_4_) showed highest oil yield 7.39 ± 0.21% and 7.58 ± 0.28% for samples, respectively (*p* ≤ 0.05). Both intensity and pretreatment time had a positive effect on extraction oil yield from sorghum grains. The combination of microwave and ultrasound pretreatments showed significant difference in oil yield than the raw sorghum grains. The highest oil yield 7.84 ± 0.31% was observed when combination of microwave power (700 W) and ultrasound intensity (60%) was applied for 30s and 10 min, respectively (*p* ≤ 0.05).

**Table 2:** Oil contents (% on DW) of sorghum at different pre-treatments before and after germination

Processing type	Growth conditions & treatments									
	Before germination					After germination				
	Raw seed	T_1_	T_2_	T_3_	T_4_	T_0_	T_1_	T_2_	T_3_	T_4_
Untreated	6.55 ± 0.25^a^	-	-	-	-	6.28 ± 0.21^b^	-	-	-	-
MW	-	7.06 ± 0.26^bc^	7.32 ± 0.29^b^	7.54 ± 0.31^ab^	7.79 ± 0.32^a^	-	6.77 ± 0.22^c^	7.02 ± 0.24^bc^	7.24 ± 0.25^b^	7.48 ± 0.27^ab^
US	-	6.79 ± 0.25^cd^	6.89 ± 0.26^c^	7.39 ± 0.21^ab^	7.58 ± 0.28^a^	-	6.53 ± 0.12^de^	6.65 ± 0.15^d^	7.15 ± 0.21^bc^	7.29 ± 0.23^b^
MW & US	-	7.10 ± 0.25^c^	7.43 ± 0.27^b^	7.61 ± 0.28^ab^	7.84 ± 0.31^a^	-	6.81 ± 0.16^d^	7.14 ± 0.19^c^	7.34 ± 0.22^bc^	7.57 ± 0.26^ab^

*Values are mean ± SEM (n = 3). Values in same row within each processing parameter with different letters were significantly different from each other (p ≤ 0. 05)*

*DW basis: Dry weight basis; T*_*0*_ *: Control germinated sample;*

*T*_*1*_ *, T*_*2*_ *, T*_*3*_ *, T*_*4*_ *: Different treatments*

*MW: Microwave processing; US: Ultrasonic processing*

*MW & US: Microwave & ultrasonic combined processing*

The raw sorghum contained palmitic (13.73 ± 0.10%), stearic (1.07 ± 0.04%) and arachidic acid (0.13 ± 0.01%) (Table 3). The difference in the fatty acid composition of sorghum oil was observed between an untreated sample and a MW-treated sample. Palmitic, stearic and arachidic acids were found 13.76 ± 0.08%, 1.11 ± 0.11% and 0.15 ± 0.03% for MW pretreatment seeds (T4), respectively (*p* ≤ 0.05). Similarly, the ultrasonic intensity and pretreatment time also showed effect on the fatty acid percentage. The highest fatty acid % for palmitic, stearic and arachidic acid was 13.75 ± 0.07%, 1.11 ± 0.09% and 0.15 ± 0.03% at 60% US intensity for 10 min (T_4_), respectively (*p* ≤ 0.05). The increase in pretreatment time improved fatty acid composition, however, pretreatment time of 5 min did not significantly affect. The change of 1.13 ± 0.10% in stearic acid was observed in case of applying combination of microwave and sonication treatments while there was no significant change in palmitic and arachidic acid. The result showed that the composition of saturated fatty acid in germinated grain was improved than ungerminated grains. The germinated grains (T_4_) showed high results of palmitic, stearic and arachidic acids. For the saturated fatty acids (SFA), palmitic acid was the most dominant fatty acids (14.56 ± 0.12%), while arachidic acid was the least (0.21 ± 0.01%) (*p* ≤ 0.05).

**Table 3:** Saturated fatty acids (% of total oils) of sorghum at different pre-treatments before and after germination

Processing type	Growth conditions & treatments									
	Before germination					After germination				
	Raw seed	T_1_	T_2_	T_3_	T_4_	T_0_	T_1_	T_2_	T_3_	T_4_
Palmitic acid (C_16:0_)										
Untreated	13.73 ± 0.10^b^	-	-	-	-	14.51 ± 0.12^a^	-	-	-	-
MW	-	13.73 ± 0.05^b^	13.73 ± 0.05^b^	13.74 ± 0.07^b^	13.76 ± 0.08^b^	-	14.51 ± 0.12^a^	14.50 ± 0.09^a^	14.52 ± 0.09^a^	14.55 ± 0.13^a^
US	-	13.70 ± 0.05^b^	13.72 ± 0.03^b^	13.74 ± 0.05^b^	13.75 ± 0.07^b^	-	14.49 ± 0.10^a^	14.50 ± 0.05^a^	14.51 ± 0.07^a^	14.54 ± 0.09^a^
MW & US	-	13.74 ± 0.10^b^	13.74 ± 0.06^b^	13.75 ± 0.05^b^	13.77 ± 0.09^b^	-	14.50 ± 0.10^a^	14.52 ± 0.06^a^	14.54 ± 0.06^a^	14.56 ± 0.12^a^
Stearic acid (C_18:0_)										
Untreated	1.07 ± 0.04^b^	-	-	-	-	1.73 ± 0.05^a^	-	-		-
MW	-	1.07 ± 0.04^b^	1.08 ± 0.04^b^	1.11 ± 0.05^b^	1.11 ± 0.05^b^	-	1.73 ± 0.10^a^	1.75 ± 0.10^a^	1.77 ± 0.11^a^	1.77 ± 0.11^a^
US	-	1.08 ± 0.06^b^	1.08 ± 0.05^b^	1.10 ± 0.02^b^	1.11 ± 0.09^b^	-	1.74 ± 0.05^a^	1.75 ± 0.10^a^	1.76 ± 0.05^a^	1.78 ± 0.10^a^
MW & US	-	1.08 ± 0.06^b^	1.09 ± 0.07^b^	1.11 ± 0.09^b^	1.13 ± 0.10^b^	-	1.74 ± 0.05^a^	1.76 ± 0.09^a^	1.77 ± 0.11^a^	1.79 ± 0.12^a^
Arachidic acid (C_20:0_)										
Untreated	0.13 ± 0.01^b^	-	-	-	-	0.18 ± 0.02^a^	-	-		-
MW	-	0.13 ± 0.01^b^	0.13 ± 0.01^b^	0.14 ± 0.02^b^	0.15 ± 0.03^b^	-	0.18 ± 0.02^a^	0.19 ± 0.03^a^	0.19 ± 0.03^a^	0.21 ± 0.01^a^
US	-	0.13 ± 0.01^b^	0.13 ± 0.02^b^	0.15 ± 0.03^b^	0.15 ± 0.03^b^	-	0.17 ± 0.02^a^	0.18 ± 0.04^a^	0.18 ± 0.04^a^	0.20 ± 0.03^a^
MW & US	-	0.14 ± 0.01^b^	0.14 ± 0.01^b^	0.14 ± 0.02^b^	0.15 ± 0.02^b^	-	0.17 ± 0.03^a^	0.18 ± 0.03^a^	0.19 ± 0.04^a^	0.20 ± 0.04^a^

*Values are mean ± SEM (n = 3). Values in same row within each processing parameter with different letters were significantly different from each other (p ≤ 0. 05)*

*T*_*0*_ *: Control germinated sample;*

*T*_*1*_ *, T*_*2*_ *, T*_*3*_ *, T*_*4*_ *: Different treatments*

*MW: Microwave processing; US: Ultrasonic processing*

*MW & US: Microwave & ultrasonic combined processing*

Table 4 presents sorghum unsaturated fatty acid before and after germination by applying different pretreatments. The results indicated that raw sorghum contained palmitoleic (0.43 ± 0.02%), oleic (37.15 ± 0.10%), linoleic (43.33 ± 0.21%), linolenic (1.55 ± 0.04%) and eicosenoic acid (0.37 ± 0.02%), respectively. Fatty acid compositions of oils did not change much with microwave and ultrasound treatment. The results show that the major fatty acids in sorghum seed oils were linoleic acid (43.53 ± 0.30%) and oleic acid (37.17 ± 0.15%) for microwave treatments, respectively. The ultrasound processed sorghum samples contained maximum amount of linolenic acid 1.60 ± 0.09% while amount of eicosenoic acid decreased from 0.37 ± 0.02% to 0.31 ± 0.01%, respectively (*p* ≤ 0.05). The eicosenoic acid increased from 0.35 ± 0.02% to 0.40 ± 0.04% as a result of applying microwave and sonication combined treatments while there was no significant change in other fatty acids (*p* ≥ 0.05). The germination caused marked changes in the percentage of palmitoleic acid (0.49 ± 0.05%), linolenic acid (1.96 ± 0.12%) and eicosenoic acid (0.39 ± 0.04%), respectively (Table 4). While the maximum decrease in unsaturated fatty acid was observed in oleic acid (34.04 ± 0.10%) (*p* ≤ 0.05) which may be due to its decomposition by the lipolytic enzymes.

**Table 4:** Unsaturated fatty acids (% of total oils) of sorghum at different pre-treatments before and after germination

Processing type	Growth conditions & treatments									
	Before germination					After germination				
	Raw seed	T_1_	T_2_	T_3_	T_4_	T_0_	T_1_	T_2_	T_3_	T_4_
Palmitoleic acid (C_16:1_)										
Untreated	0.43 ± 0.02^b^	-	-	-	-	0.48 ± 0.02^a^	-	-	-	-
MW	-	0.43 ± 0.01^b^	0.43 ± 0.02^b^	0.43 ± 0.02^b^	0.44 ± 0.04^b^	-	0.47 ± 0.01^a^	0.48 ± 0.02^a^	0.48 ± 0.02^a^	0.49 ± 0.05^a^
US	-	0.43 ± 0.02^b^	0.43 ± 0.02^b^	0.44 ± 0.03^b^	0.45 ± 0.05^b^	-	0.47 ± 0.01^a^	0.47 ± 0.01^a^	0.48 ± 0.03^a^	0.48 ± 0.04^a^
MW & US	-	0.43 ± 0.02^b^	0.44 ± 0.03^b^	0.45 ± 0.04^b^	0.45 ± 0.05^b^	-	0.47 ± 0.01^a^	0.48 ± 0.02^a^	0.49 ± 0.04_a_	0.49 ± 0.05^a^
Oleic acid (C_18:1_)										
Untreated	37.15 ± 0.10^a^	-	-	-	-	34.1 ± 0.11^b^	-	-	-	-
MW	-	37.12 ± 0.11^a^	37.15 ± 0.11^a^	37.15 ± 0.13^a^	37.17 ± 0.15^a^	-	34.04 ± 0.10^b^	34.1 ± 0.10^b^	34.10 ± 0.11^b^	34.12 ± 0.14^b^
US	-	37.15 ± 0.13^a^	37.15 ± 0.13^a^	37.18 ± 0.11^a^	37.20 ± 0.14^a^	-	34.08 ± 0.12^b^	34.11 ± 0.12^b^	34.11 ± 0.10^b^	34.15 ± 0.13^b^
MW & US	-	37.17 ± 0.14^a^	37.17 ± 0.14^a^	37.20 ± 0.15^a^	37.20 ± 0.14^a^	-	34.12 ± 0.13^b^	34.14 ± 0.10^b^	34.15 ± 0.11^b^	34.15 ± 0.11^b^
Linoleic acid (C_18:2_)										
Untreated	43.33 ± 0.21^a^	-	-	-	-	43.45 ± 0.22^a^	-	-		-
MW	-	43.39 ± 0.26^a^	43.45 ± 0.29^a^	43.47 ± 0.29^a^	43.53 ± 0.30^a^	-	43.48 ± 0.24^a^	43.52 ± 0.26^a^	43.55 ± 0.26^a^	43.57 ± 0.31^a^
US	-	43.33 ± 0.21^a^	43.34 ± 0.20^a^	43.46 ± 0.27^a^	43.46 ± 0.28^a^	-	43.42 ± 0.21^a^	43.42 ± 0.21^a^	43.47 ± 0.25^a^	43.48 ± 0.25^a^
MW & US	-	43.42 ± 0.23^a^	43.44 ± 0.23^a^	43.47 ± 0.29^a^	43.47 ± 0.30^a^	-	43.45 ± 0.22^a^	43.46 ± 0.22^a^	43.57 ± 0.29^a^	43.57 ± 0.31^a^
Linolenic acid (C_18:3_)										
Untreated	1.55 ± 0.04^b^	-	-	-	-	1.89 ± 0.07^a^	-	-		-
MW	-	1.54 ± 0.04^d^	1.54 ± 0.04^d^	1.56 ± 0.05^cd^	1.58 ± 0.06^c^	-	1.85 ± 0.05^bc^	1.85 ± 0.05^bc^	1.91 ± 0.10^b^	1.93 ± 0.11^ab^
US	-	1.55 ± 0.04^d^	1.56 ± 0.05^cd^	1.59 ± 0.06^c^	1.60 ± 0.09^c^	-	1.86 ± 0.05^bc^	1.86 ± 0.08^bc^	1.94 ± 0.12^ab^	1.96 ± 0.12^a^
MW & US	-	1.55 ± 0.05^d^	1.57 ± 0.06^cd^	1.58 ± 0.08^c^	1.59 ± 0.08^c^	-	1.87 ± 0.06^bc^	1.90 ± 0.01^b^	1.93 ± 0.11^ab^	1.96 ± 0.12^a^
Eicosenoic acid (C_20:1_)										
Untreated	0.37 ± 0.02^a^	-	-	-	-	0.34 ± 0.01^b^	-	-		-
MW	-	0.37 ± 0.02^ab^	0.37 ± 0.02^ab^	0.39 ± 0.02^a^	0.40 ± 0.03^a^	-	0.34 ± 0.02^b^	0.35 ± 0.01^b^	0.35 ± 0.02^b^	0.38 ± 0.03^a^
US	-	0.31 ± 0.01^bc^	0.32 ± 0.01^bc^	0.35 ± 0.01^b^	0.35 ± 0.02^b^	-	0.3 ± 0.01^c^	0.3 ± 0.01^c^	0.32 ± 0.02^bc^	0.33 ± 0.03^bc^
MW & US	-	0.35 ± 0.02^b^	0.37 ± 0.03^ab^	0.40 ± 0.03^a^	0.40 ± 0.04^a^	-	0.33 ± 0.02^bc^	0.34 ± 0.01^b^	0.39 ± 0.04^a^	0.39 ± 0.04^a^

Values are mean ± SEM (*n* = 3). Values in same row within each processing parameter with different letters were significantly different from each other (*p* ≤ 0. 05)

*T*_*0*_ *: Control germinated sample;*

*T*_*1*_ *, T*_*2*_ *, T*_*3*_ *, T*_*4*_ *: Different treatments*

*MW: Microwave processing; US: Ultrasonic processing*

*MW & US: Microwave & ultrasonic combined processing*

Table 5 shows percentage of saturated fatty acids (SFA), unsaturated fatty acids (Un-SFA) and Un-SFA/SFA ratio before and after germination by applying different pre-treatments. The results indicated that ungerminated sorghum oil contained 14.93–15.05% and 82.83–83.12% of SFA and Un-SFA, respectively. After germination, the percentage of saturated fatty acids increased 16.4–16.55% while decreased for unsaturated fatty acids 80.13–80.56% was observed (*p* ≤ 0.05).

**Table 5:** Percentage of saturated fatty acids (SFA), unsaturated fatty acids (Un-SFA) and Un-SFA/SFA ratio of sorghum at different pre-treatments before and after germination

Processing type	Growth conditions & treatments									
	Before germination					After germination				
	Raw seed	T_1_	T_2_	T_3_	T_4_	T_0_	T_1_	T_2_	T_3_	T_4_
Saturated fatty acids										
Untreated	14.93^b^	-	-	-	-	16.42^a^	-	-	-	-
MW	-	14.93^b^	14.94^b^	14.99^b^	15.02^b^	-	16.42^a^	16.44^a^	16.48^a^	16.53^a^
US	-	14.91^b^	14.93^b^	14.99^b^	15.01^b^	-	16.4^a^	16.43^a^	16.45^a^	16.52^a^
MW & US	-	14.96^b^	14.97^b^	15.0^b^	15.05^b^	-	16..41^a^	16.46^a^	16.5^a^	16.55^a^
Unsaturated fatty acids										
Untreated	82.83^a^	-	-	-	-	80.26^b^	-	-	-	-
MW	-	82.85^a^	82.94^a^	83.0^a^	83.12^a^	-	80.18^b^	80.3^b^	80.39^b^	80.49^b^
US	-	82.77^a^	82.8^a^	83.02^a^	83.06^a^	-	80.13^b^	80.16^b^	80.32^b^	80.4^b^
MW & US	-	82.94^a^	82.99^a^	83.1^a^	83.11^a^	-	80.24^b^	80.32^b^	80.53^b^	80.56^b^
Un-SFA/SFA ratio										
Untreated	5.54^a^	-	-	-	-	4.88^b^	-	-	-	-
MW	-	5.54^a^	5.55^a^	5.53^a^	5.53^a^	-	4.88^b^	4.88^b^	4.87^b^	4.86^b^
US	-	5.55^a^	5.54^a^	5.53^a^	5.53^a^	-	4.88^b^	4.87^b^	4.88^b^	4.86^b^
MW & US	-	5.54^a^	5.54^a^	5.54^a^	5.52^a^	-	4.88^b^	4.87^b^	4.88^b^	4.86^b^

*Values are mean ± SEM (n = 3). Values in same row within each processing parameter with different letters were significantly different from each other (p ≤ 0. 05)*

*T*_*0*_ *: Control germinated sample; T*_*1*_ *, T*_*2*_ *, T*_*3*_ *, T*_*4*_ *: Different treatments*

*MW: microwave processing; US: ultrasonic processing*

*MW & US: Microwave & ultrasonic combined processing*

*SFA: Saturated fatty acids; Un-SFA: Unsaturated fatty acids*

## Discussion

### Oils contents of sorghum grains before and after germination

The results of oil extraction yield are in agreement with previous findings [23–25]. By using microwave radiation in oil seeds, a higher extraction yield and an increase in mass transfer coefficients can be obtained because the cell membrane is more severely ruptured. Apart from this, permanent pores are generated as accordingly and this enables the oil to move through permeable cell walls [26]. Similarly, different research studies found increased in oil yield with high ultrasound amplitude level [27, 28]. This increase in yield for ultrasound treatment has been proposed due to the effect of ultrasonic cavitation. The physical effects of cavitation immediately disrupt intact biological cells in the hot spots by rupturing biological membranes and cell walls. Thus, cellular material pours out into the liquid medium made up of the solvent and lipids are selectively dissolved in it. This process forms the basis for ultrasound-assisted solvent extraction and is responsible for the much higher oil yields from ultrasonic treatment in comparison with other methods [29, 30]. The combination of ultrasound pretreatment with other physical methods could weaken the particle surface bonds and enhance the extraction yield effectively [31]. In the present study, germination significantly decreased crude oil content of raw and pretreated sorghum flours, which was in agreement with results of previous investigation [18, 32, 33]. The reduction may be due to the fact that biochemical and physiological changes occurred during germination and such changes required energy to proceed and therefore part of the seed oil was utilized for the production of this energy. The observed decrease in fat content of sorghum flour during germination might be attributed to the increased activities of the lipolytic enzymes during germination, which hydrolyze fats to fatty acids and glycerol [18, 33]. From a nutritional point of view, food-grade sorghum flour turns out to be a very interesting product. In fact, its nutritional value is comparable to those belonging to the ordinary flours obtained from the noble cereals [34, 35].

### Sorghum saturated fatty acids (SFA) before and after germination

The fatty acid composition of sorghum seed is very similar to previous reported composition [36, 37]. Yoshida et al. [38], Anjum et al. [39] and Yoshida et al. [40] studied the effect of microwave treatment on peanut seeds (*Arachis hypogaea* L.), sunflower seed (*Heliantus annuus* L.) and pumpkin seeds (*Cucurbita spp.*), respectively. These authors reported a change in the fatty acid composition of vegetable oils through the effect of microwave treatment. Changes in the fatty acid composition after MW pretreatment of oilseeds have been reported by Yoshida et al. [41]. The findings of US treatments were in agreement with those reported by Luque–Garcia and Luque de Castro [42]. The slight increase might be due to non-conversion of free fatty acids to carbohydrates which may lead to increase in fat composition during germination [43]. The increased intake of saturated fatty acids leads towards the risks of cardiovascular diseases, cancer and autoimmune disorders [44]. Palmitic acid and stearic acids are some of the main fatty acids present in animals, vegetables and human milk fats. Several controversies are there about health and adverse impact of palmitic and stearic acid on human health, particularly about role of palmitic acid in cardiovascular disease and carcinogenicity [45, 46].

### Sorghum unsaturated fatty acid (un-SFA) before and after germination

The results for unsaturated fatty acids present in sorghum grains are well correlated with previous investigations [36, 37]. Seed oils undergo changes in terms of chemical and physical properties when they interact with the food or the atmosphere. The fatty acid composition of oil can be an indicator of its stability, physical properties and nutritional value. Some food processing techniques can affect fatty acid composition of oils when hardly subjected to successive heating [42, 47, 48]. Cravotto et al. [49] reported the non-significant changes in polyunsaturated fatty acids of seaweed oil obtained under conventional and ultrasonic conditions. Kang et al. [50] mentioned that oleic acid was decreased by 50%, whereas those of linoleic and linolenic acid were increased by 1.3 and 5.4 times, respectively after 7 days of germination. Hahm et al. [51] reported the germinated derooted sesame rich in linolenic acid. Some studies suggested that linolenic acid intakes reduce the risk of cardiovascular diseases [52, 53]. An interaction between dietary linolenic acid intake and cardiovascular health in humans was reviewed [54, 55].

### Percentage of saturated fatty acids (SFA), unsaturated fatty acids (un-SFA) and un-SFA/SFA ratio

The polyunsaturated fatty acids in most of sorghum varieties were found higher than monounsaturated fatty acids [36]. The white sorghum oil contained 12.40% total saturated fatty acid and 87.60% total unsaturated fatty acid, respectively [56]. The unsaturated fatty acids level of sorghum decreased on germination. The observed decrease might be due to the increased activities of the lipolytic enzymes during germination, which hydrolyze oils to fatty acids and glycerol [57]. The simpler products can be used for synthesis of carbohydrate and protein or as a source of energy for developing embryo. Similar observation was made by other researchers [58]. Oils being source of lipids, are of more nutritional value if they have more unsaturated to saturated fatty acid ratio [59].

## Conclusions

Lipids composition due to their pharmacological significance has caught the attention of both consumer and industries. The presence of all saturated and unsaturated fatty acids essential for human health in sorghum oil could be alternative source of edible oil. It can be concluded that the germination of sorghum grains caused marked reduction in oil content. Moreover, also showed a decrease in total unsaturated fatty acids while the total saturated fatty acids increased by germination of seeds. Fatty acid analysis of sorghum oil suggested that pre-treatment strategies will not affect the quality of the oil with respect to essential fatty acid content. Every pre-treatment has some positive and negative impact on the quality and character of the extracted oils. Thus, selection of appropriate pre-treatment strategies will help to achieve enhanced seed oil with desired quality. The results of present study suggested that combination of microwave and ultrasonic treatment was best method for extracting high quality sorghum oil.
